# The CREB-miR-9 Negative Feedback Minicircuitry Coordinates the Migration and Proliferation of Glioma Cells

**DOI:** 10.1371/journal.pone.0049570

**Published:** 2012-11-20

**Authors:** Xiaochao Tan, Shan Wang, Bin Yang, Liyuan Zhu, Bin Yin, Tengfei Chao, Jizong Zhao, Jiangang Yuan, Boqin Qiang, Xiaozhong Peng

**Affiliations:** 1 State Key Laboratory of Medical Molecular Biology, Institute of Basic Medical Sciences, Chinese Academy of Medical Sciences and Peking Union Medical College, Beijing, China; 2 Department of Oncology, Tongji Hospital, Tongji Medical College, Huazhong University of Science and Technology, Wuhan, China; 3 The Department of Neurosurgery, Beijing Tiantan Hospital, Beijing, China; Sun Yat-sen University Medical School, China

## Abstract

Migration-proliferation dichotomy is a common mechanism in gliomagenesis; however, an understanding of the exact molecular mechanism of this “go or grow” phenomenon remains largely incomplete. In the present study, we first found that microRNA-9 (miR-9) is highly expressed in glioma cells. MiR-9 inhibited the proliferation and promoted the migration of glioma cells by directly targeting cyclic AMP response element-binding protein (CREB) and neurofibromin 1 (NF1), respectively. Our data also suggested a migration-inhibitory role for CREB through directly regulating the transcription of NF1. Furthermore, we found that the transcription of miR-9-1 is under CREB's control, forming a negative feedback minicircuitry. Taken together, miR-9 inhibits proliferation but promotes migration, whereas CREB plays a pro-proliferative and anti-migratory role, suggesting that the CREB-miR-9 negative feedback minicircuitry plays a critical role in the determination of “go or grow” in glioma cells.

## Introduction

Although a great deal of progress has been made over the past few decades in modeling cancer growth and progression, our knowledge of malignant glioma, the most common diffusive primary brain tumor, is still incomplete [1], [2]. Gliomas are able to not only proliferate but also invade the surrounding brain tissue, leading to very poor prognoses for patients suffering from gliomas [3]. Chemotherapeutic agents generally have little efficacy against gliomas [4]. The limited treatment options for glioma have therefore led us to investigate the genetic changes underlying this deadly cancer. It is widely believed that mutations trigger the switch from a proliferative to motile phenotype of cancer cells. Initially, the cancer cells obtain mutations altering the control of proliferation, thereby leading to uncontrolled cell division [5]. Accumulating mutations then result in the emergence of phenotypes characterized by high motility and angiogenesis. However, these mutation-driven phenotypic changes alone are not sufficient to explain the fast evolution and rapid adaptation that are characteristic of gliomas. Increasing experimental evidence suggests that the proliferation rate of migratory glioma cells is low compared with cells in the tumor core, indicating an inverse correlation between mobility and proliferation of the cell population [4], [6]. A migration-proliferation dichotomy was employed to evaluate this phenomenon in which proliferative and migratory tumor cells are mutually exclusive phenotypes [7]. More and more evidence supports this theory, and some reports suggest that a single gene can coordinate the proliferation and migration of the glioma cells [8]–[10]. However, the molecular mechanism of migration-proliferation dichotomy deserves further investigation.

The brain-enriched microRNA-9 (miR-9) has been implicated in nervous system development and physiological and pathological processes in several organisms [11]. Loss of miR-9 suppresses proliferation but promotes the migration of human neural progenitor cells cultured in vitro [8]. The expression patterns and roles of miR-9 are diverse in different types of cancers: in some types of tumors, such as neuroblastoma [12], medulloblastoma [13] and ovarian cancer [14], miR-9 is down-regulated and functions as a tumor suppressor; in other tumors, including colorectal [15] and breast cancers [16], the highly expressed miR-9 promotes the growth and/or metastasis of the cancer cells. Chao et al. proposed a proliferation-inhibitory role of the highly expressed miR-9 in T98G cells [17], and recently, Schraivogel et al. found that miR-9/miR-9* promotes neurosphere formation of glioblastoma stem cells through targeting of the tumor suppressor CAMTA1 [18]. Although shown to correlate with glioblastoma progression [19], the role of miR-9 in gliomagenesis is still poorly understood.

The over-expression of cyclic AMP response element-binding protein (CREB) in malignancies implies an oncogenic role [20]–[23]. As our previous study described, CREB is highly expressed in glioma tissues and cell lines and dramatically contributes to the growth and survival of glioma cells in vitro and in vivo [24]. In one report, the expression of miR-9-2 was shown to be under CREB's control during neuronal differentiation [25], and the possibility of the regulation of miR-9 by CREB was predicted by Wu et al. several years ago [26]. These findings provide clues that CREB might contribute to the expression of miR-9 in glioma cells.

In this study, we investigated the roles of miR-9 and evaluated if CREB modulates the expression of miR-9 in glioma cells. Interestingly, we also identified CREB as a novel target of miR-9, suggesting a minicircuitry involving CREB and miR-9-1 in the coordination of migration and proliferation of glioma cells.

## Results

### MiR-9 is highly expressed in glioma cells

**Figure 1:**
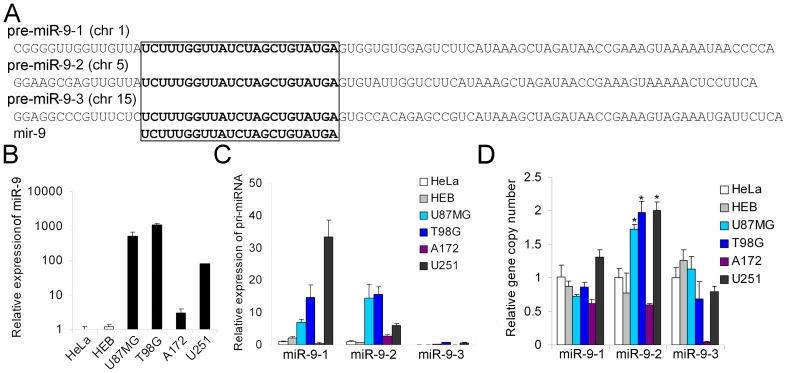
MiR-9 is highly expressed in glioma cell lines. (A) Schematic representation showing that miR-9 can be generated by the processing of any of the three primary transcripts encoded by three distinct genes (miR-9-1, miR-9-2 and miR-9-3). (B and C) The expression levels of mature miR-9 as well as pri-miR-9-1, pri-miR-9-2 and pri-miR-9-3 were determined in the human cervical carcinoma cell line (HeLa), normal human glial cell line (HEB) and four glioma cell lines (U87MG, T98G, A172 and U251) by quantitative RT-PCR (mean ± SD, n = 3). (D) Genomic DNA was extracted from the six cell lines (HeLa, HEB, U87MG, T98G, A172 and U251), and the gene copy numbers of miR-9-1, miR-9-2 and miR-9-3 were determined by quantitative real-time PCR (mean ± SD, n = 3). *, P<0.05, two-tailed unpaired Student's t test, relative to HEB and HeLa.

MiR-9 is a brain-enriched miRNA that can be generated by three distinct genes (miR-9-1, miR-9-2 and miR-9-3) (Fig. 1A). By quantitative RT-PCR, we found that miR-9 is highly expressed in four glioma cell lines (U87MG, T98G, A172 and U251) compared with HeLa cells or the normal human glial cell line HEB (Fig. 1B). We also found that the expression levels of primary microRNA-9-1 (pri-miR-9-1) and pri-miR-9-2 are high in U87MG, T98G and U251 but not in A172 and that the expression level of pri-miR-9-3 is extremely low in all six cell lines (Fig. 1C). The aberrant hypermethylation of miR-9-3, which has been reported in NSCLC and breast cancer [27], [28], might be one of the reasons why its expression is remarkably inhibited. Gene copy number amplifications often contribute to high gene expression; therefore, we determined the copy numbers of miR-9-1, miR-9-2 and miR-9-3 in the six cell lines. Interestingly, we found a significant amplification of miR-9-2 (but not miR-9-1 or miR-9-3) gene copy number in all glioma cell lines except A172 (Fig. 1D), suggesting that the copy number amplification of miR-9-2 might contribute to the high expression level of miR-9 in glioma cells.

### MiR-9 inhibits growth but stimulates migration of glioma cells

**Figure 2:**
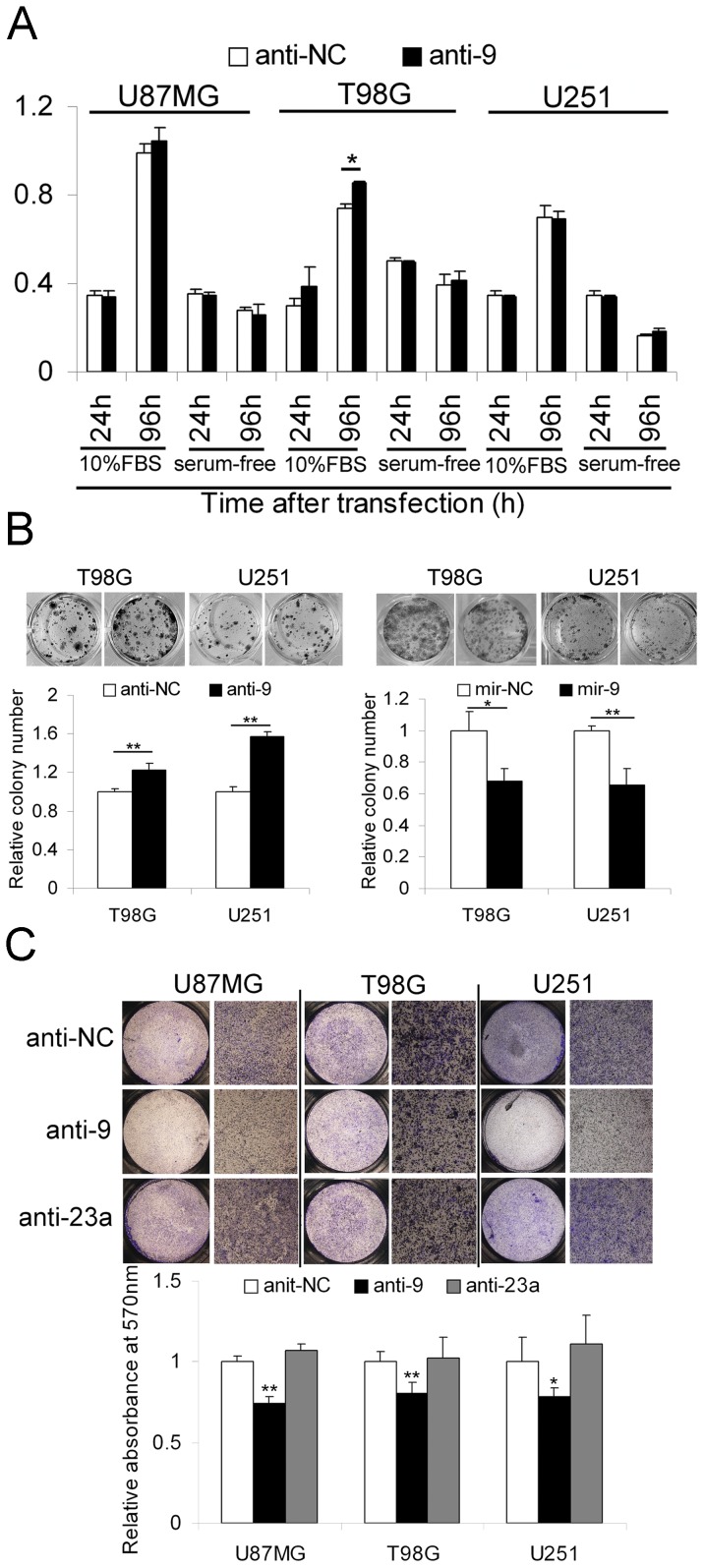
The effects of miR-9 knockdown on glioma cell growth, colony formation and migration. (A) MTT assays were performed to evaluate the effects of miR-9 knockdown on the growth and survival of glioma cells (U87MG, T98G and U251). Data are represented as the mean ± SD, n = 4. (B) Colony formation assays were utilized to test the effects of knocking down and over-expressing miR-9 on the colony formation ability of T98G and U251 cells. Data are represented as the mean ± SD, n = 4. (C) Transwell migration assays were employed to evaluate the effects of knocking down miR-9 and miR-23a (as a negative control) on the migration of glioma cells. On the top are representative photographs of transwell assays. The bound crystal violet staining was released with 33% glacial acetic acid and quantified by absorbance measurement (OD570-630) (mean ± SD, n = 4). *, *P*<0.05, **, *P*<0.01, two-tailed unpaired Student's t test.

Although miR-9 has been reported to be progression-associated in glioblastoma and is highly expressed in the glioma cell lines in our study, its function remains largely unknown. Chao et al. found that miR-9 inhibits the proliferation of T98G glioma cells [17]. This highly expressed miR-9 was, however, expected to contribute to the malignancy of glioma cells. Because miR-9 was reported to be involved in the metastasis of breast cancer cells [16], the effects of knocking down miR-9 on cell growth, survival, colony formation and migration were evaluated. The results of MTT assays showed that miR-9 knockdown promotes the growth of T98G cells but has little effect on the growth of U87MG and U251 cells or the survival of the three glioma cell lines following transfection with miR-9 antagomirs for 4 days (Fig. 2A). However, knocking down miR-9 significantly promoted the colony formation abilities of T98G and U251 cells. Conversely, elevating the level of miR-9 could suppress the colony growth of T98G and U251 cells (Fig. 2B). To comprehensively evaluate the role of miR-9 in glioma cell migration, transwell migration assays and scratch wound healing assays were employed. The results of transwell migration assays showed that knocking down miR-9 significantly inhibited the migration of the glioma cells (U87MG, T98G and U251), suggesting a migration-enhancive role for miR-9 (Fig. 2C). Moreover, the scratch wound healing assay confirmed that miR-9 knockdown slowed the migration of glioma cells (Fig. S1).

### MiR-9 inhibits proliferation by targeting CREB

**Figure 3:**
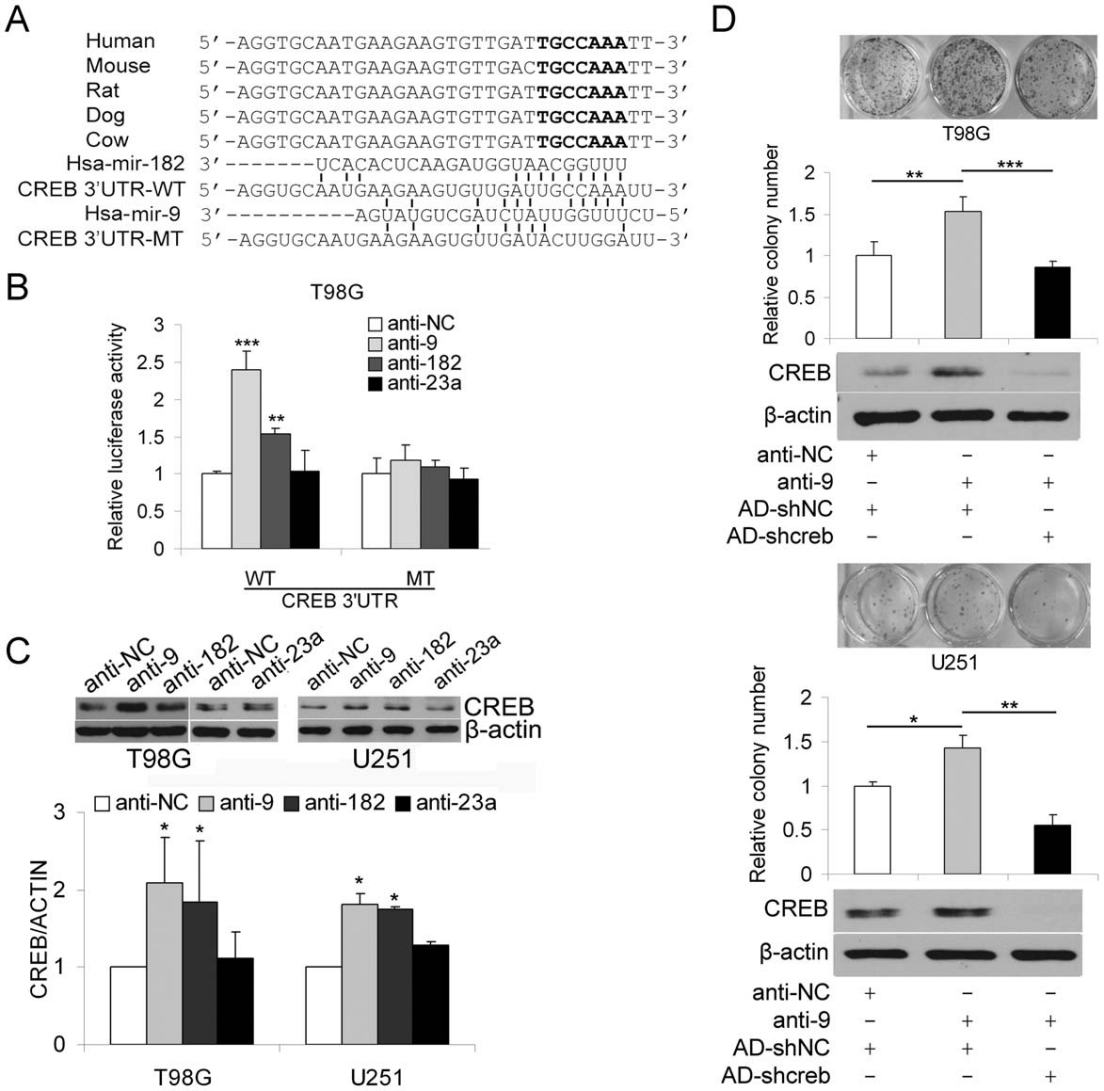
MiR-9 inhibits proliferation by targeting CREB. (A) MiR-9 and miR-182 share a binding site on the 3′UTR of CREB. A putative miR-182 binding site that is highly conserved among mammals was predicted by three algorithms (TargetScan, PicTar and MiRDB). A mutated CREB 3′UTR (CREB 3′UTR-MT) was generated by introducing mutations into the miR-182 binding site. (B) Luciferase reporter plasmids containing the wild-type (WT) or mutated (MT) CREB 3′UTR were co-transfected with synthetic miR-9, miR-182 or control miRNA mimics. After 24 h, the cells were harvested and the normalized luciferase activity was determined (mean ± SD, n = 4). (C) T98G cells were transfected with synthetic mimics of miR-9, miR-23a, miR-182 or control miRNA mimics. After 48 h, total cellular protein was extracted and the protein level of CREB was detected by western blotting (top). The western blotting results were quantified and plotted (mean ± SD, n = 3) (bottom). *, *P*<0.05; **, *P*<0.01, two-tailed unpaired Student's t test. (D) T98G and U251 cells were transfected with miR-9 or control antagomirs followed by infection with AD-shcreb or AD-shNC. After 48 h, the transfected/infected cells were subjected to colony formation assay. The colonies were counted and plotted (mean ± SD, n = 4). On the top are representative photographs of the cell colonies, and the bottom depicts Western blotting results showing the CREB protein level of the transfected/infected glioma cells. *, P<0.05; **, P<0.01; ***, P<0.001, two-tailed unpaired Student's t test.

We have confirmed both anti-proliferative and pro-migratory roles of miR-9 in glioma cells; however, the functional downstream targets of miR-9 needed to be elucidated. In a recent report, miR-182 was shown to target CREB in gastric adenocarcinoma cells [29]. Interestingly, in our study, we found that the binding site of miR-182 in the 3′UTR of CREB also has the potential to interact with miR-9 (Fig. 3A). Knocking down miR-9 and miR-182 significantly increased the luciferase activity of the CREB 3′UTR reporter (but not the CREB 3′UTR reporter containing a mutated miR-182 binding site) in T98G cells and the protein level of CREB in T98G and U251 cells. As a negative control, knockdown of miR-23a did not affect either the luciferase activity of the CREB 3′UTR reporter or the protein level of CREB (Fig. 3B, C). The expression level of miR-182 in glioma cells was similar to that of HeLa and HEB cells, suggesting that miR-9, rather than miR-182, plays a dominant role in the regulation of CREB (Fig. S2A). Importantly, knocking down miR-9 had little effect on the expression level of miR-182 in T98G cells (Fig. S2B), ruling out the possibility that miR-9 functions by regulating the expression of miR-182.

Because CREB was shown to be a proliferation enhancer in our previous study, we theorized that it could potentially mediate the anti-proliferative function of miR-9. Indeed, knocking down CREB with an adenovirus-mediated shRNA abolished the up-regulation of the protein level of CREB and the enhancement of colony growth of T98G and U251 cells caused by knocking down miR-9 (Fig. 3D), suggesting that miR-9 inhibits the colony formation ability of glioma cells at least partly through suppressing CREB.

### MiR-9 promotes migration through repression of NF1

**Figure 4:**
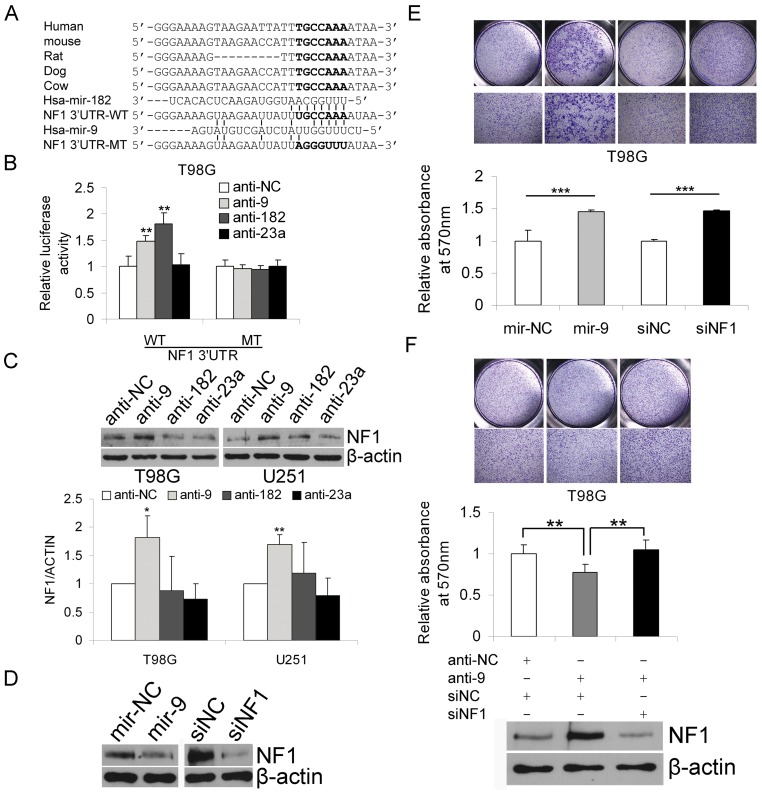
MiR-9 promotes migration through targeting NF1. (A) The NF1 3′UTR contains a putative miR-182 binding site which also has the potential to interact with miR-9. The mammalian alignment is shown on the top. A mutated NF1 3′UTR was generated by mutating the miR-182 binding site. (B) Luciferase reporter assays were performed to test the interactions between the miRNAs and the NF1 3′UTR. NF1 3′UTR luciferase reporter constructs were co-transfected with synthetic miRNA mimics (miR-9, miR-182 and miR-23a) or control mimics (miR-NC), and 24 h later the normalized luciferase activity was determined. Data are represented as the mean ± SD, n = 4. (C) MiR-9, miR-182 and miR-23a were knocked-down in T98G and U251 cells by transfection with miRNA antagomirs, and NF1 protein level was determined by western blotting and quantified by densitometric measurement (mean ± SD, n = 3). (D) T98G cells were transfected with miR-9 mimics (or control mimics, miR-NC) or siRNA targeting NF1 (or control non-specific siRNA), and whole cell protein was extracted for determination of NF1 protein levels by western blotting. (E) Over-expression of miR-9 or knockdown of NF1 enhances the migration of T98G cells in a transwell migration assay. Representative photographs are shown on the top. Crystal violet staining was removed and quantified by absorbance measurement (OD570-630) (mean ± SD, n = 4). (F) NF1 knockdown abolished the effect of decreased miR-9 on the migration of T98G cells. Crystal violet staining was removed and quantified by absorbance measurement (OD570-630) (mean ± SD, n = 4). **, *P*<0.01, ***, *P*<0.001, two-tailed unpaired Student's t test. On the bottom is a representative western blot result showing that knocking down miR-9 increased the NF1 protein level, whereas simultaneous transfection of siNF1 abolished the increased NF1 protein level.

NF1, a tumor suppressor involved in gliomagenesis, has been reported to modulate cell motility [30]–[32]. Using bioinformatic prediction, we found a putative miR-182 binding site that also had the potential to interact with miR-9 in the 3′UTR of NF1 (Fig. 4A). The results of luciferase reporter assays showed that knocking down miR-9 and miR-182 could derepress the luciferase activity of the NF1 3′UTR reporter but had no effect on the luciferase activity of the mutated NF1 3′UTR reporter in T98G cells (Fig. 4B). Knocking down miR-9 but not miR-182 in T98G and U251 cells led to an increase in the protein level of NF1 (Fig. 4C). We detected the expression of NF1 in HeLa, HEB and the four glioma cell lines (U87MG, T98G, A172 and U251) and found that, with the exception of A172 cells, the NF1 mRNA and protein levels in glioma cells were low compared with HEB cells. A172 cells expressed the lowest level of miR-9 and the highest level of NF1 mRNA and protein (Fig. S3A, B). Transfection with synthetic miR-9 mimics or siRNA against NF1 down-regulated the NF1 protein level and stimulated the migration of T98G (Fig. 4D and E) and U251 cells (Fig. S4). However, NF1 knockdown had little effect on the proliferation of T98G and U251 cells (Fig. S3C, D). In rescue experiments, NF1 knockdown restored the migration capability of T98G cells with miR-9 knocked down (Fig. 4F), suggesting that miR-9 modulates the migration capacity of glioma cells at least partly by targeting NF1.

### CREB inhibits the migration of glioma cells and regulates the transcription of *NF1*

**Figure 5:**
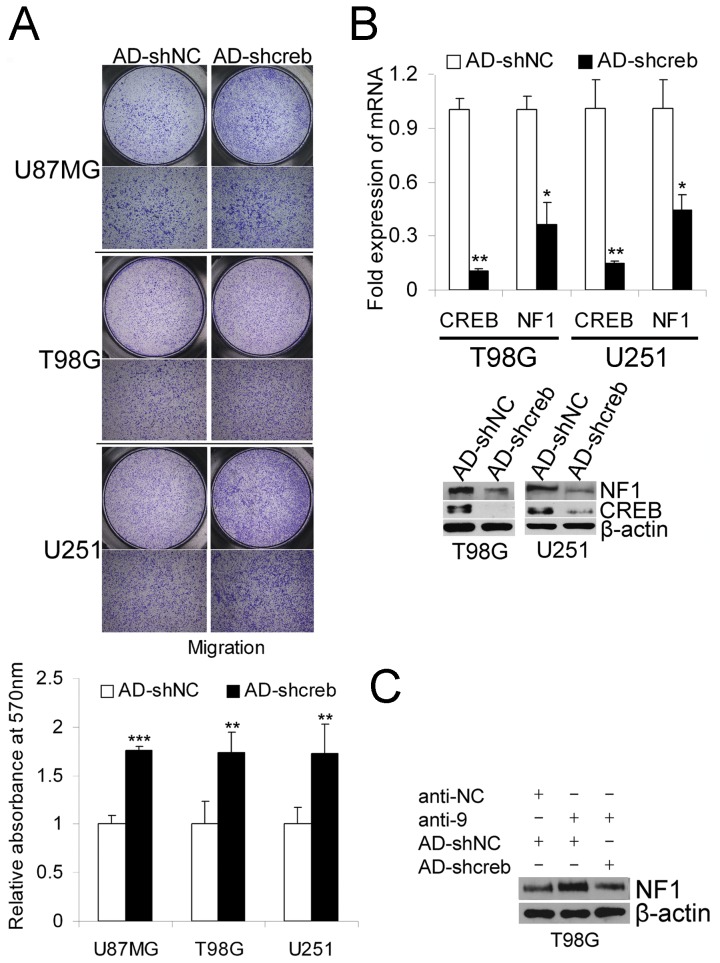
CREB regulates NF1 and knocking down CREB inhibits migration. (A) The migratory capacity of AD-shcreb or AD-shNC-infected U87MG, T98G and U251 cells were analyzed in a transwell migration assay (top). The bound crystal violet staining was released and quantified by measuring the OD570-630 (mean ± SD, n = 3) (bottom). (B) The mRNA and protein levels of NF1 were detected in control T98G and U251 cells or in cells with CREB knocked down by quantitative RT-PCR and western blotting, respectively. *, *P*<0.05; **, *P*<0.01; ***, *P*<0.001, two-tailed unpaired Student's t test. (C) T98G cells were transfected with miR-9 antagomirs or controls followed by infection of adenovirus-mediated shRNA for CREB (AD-shcreb) or AD-shNC, and the protein level of NF1 was detected by western blotting.

In our previous study, CREB was shown to promote the growth and survival of glioma cells. However, the role of CREB in the migration of glioma cells remains unknown. Unexpectedly, we found that CREB knockdown significantly promoted the migration capacity of U87MG, T98G and U251 cells (Fig. 5A) and stimulated the scratch wound healing response of U87MG and U251 cells (Fig. S5). Interestingly, NF1 was reported to be transcriptionally regulated by CREB in human cells [33], so we evaluated the NF1 mRNA and protein levels after CREB knockdown. The results of quantitative RT-PCR and western blotting showed that knocking down CREB led to significant reductions of the levels of NF1 mRNA and protein (Fig. 5B). Furthermore, ectopic expression of CREB in T98G cells enhanced the transcriptional activity of the NF1 promoter containing the wild-type CRE but not a mutated one (Fig. S6), suggesting that NF1 is directly regulated by CREB in glioma cells. We wondered whether miR-9, which can directly target CREB, could regulate NF1 by repressing CREB. Indeed, knocking down CREB the increase of NF1 protein caused by miR-9 antagomirs (Fig. 5C), suggesting an indirect regulation of NF1 by miR-9.

### MiR-9-1 is directly regulated by CREB

**Figure 6:**
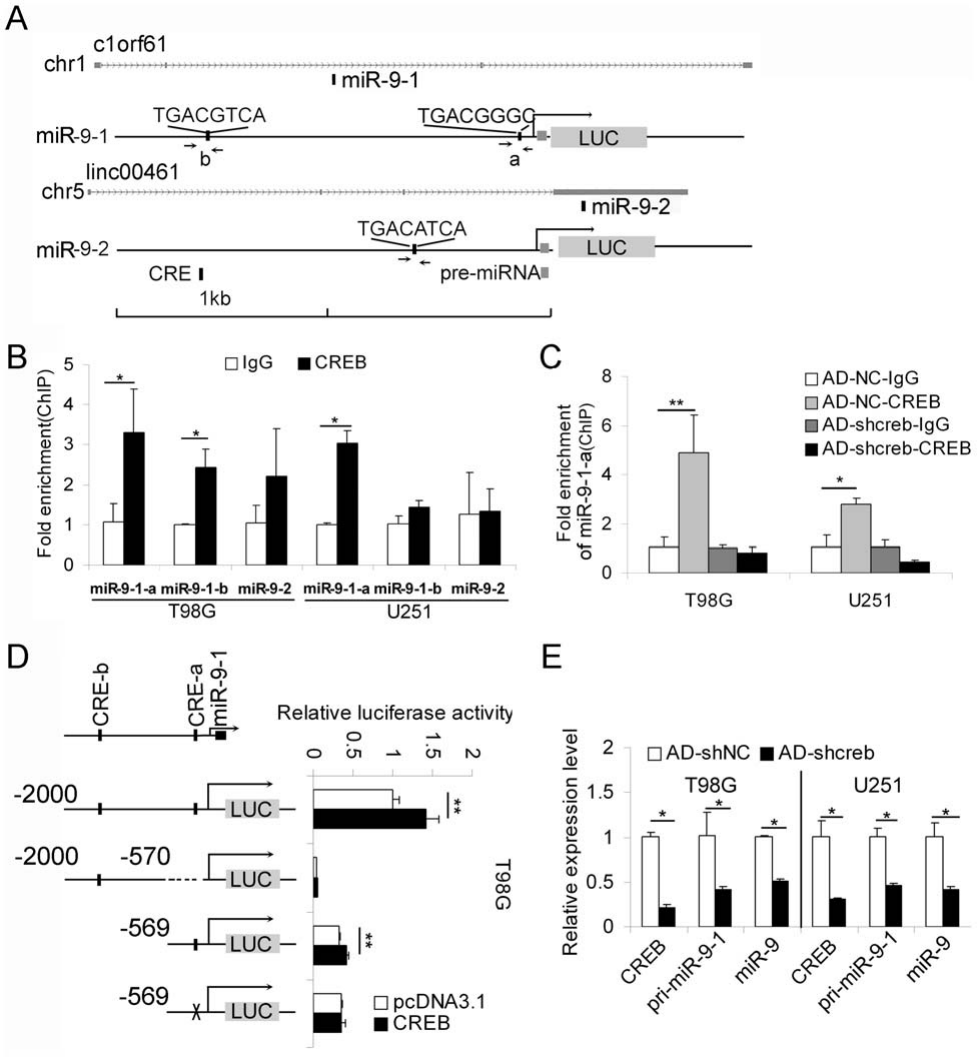
MiR-9-1 is under CREB's control. (A) Location of putative CREs within the 5′ flanking regions of miR-9-1 and miR-9-2. MiR-9-1 is located in an intron of the gene c1orf61 (chromosome 1), whereas miR-9-2 is located in an exon of linc00461 (chromosome 5). Three pairs of primers (miR-9-1-a, miR-9-1-b and miR-9-2) were designed to detect the binding capacity of CREB to the predicted CREs of miR-9-1-a, miR-9-1-b and miR-9-2, respectively, by ChIP-qPCR assays. Both the 5′ flanking sequences (2 kb) and the pre-miRNA bodies of miR-9-1 and miR-9-2 were inserted upstream of the luciferase reporter (gray box shown by LUC). The arrows denote the positions of primers used for ChIP-qPCR. (B) ChIP-qPCR assays were performed in T98G and U251 cells to detect the binding capacity of CREB to the putative CREs of miR-9-1 and miR-9-2 (mean ± SD, n = 3). (C) In AD-shNC/AD-shcreb-infected T98G and U251 cells, ChIP-qPCR was performed to detect the binding capacity of CREB on CRE-miR-9-1-a (mean ± SD, n = 3). (D) CREB enhances the transcription of miR-9-1. The 5′ flanking sequences (−2 kb+miR-9-1; −2 kb, −570 bp; −560 bp+miR-9-1) without mutations or with a mutation of CRE-a (−569+miR-9-1, from TGACGGGC to TGGAGGGC) in miR-9-1 were inserted upstream of the luciferase cassette. The luciferase reporter constructs were co-transfected with CREB expression plasmids or control vectors and the normalized luciferase activity was determined (mean ± SD, n = 4). (E) The mRNA expression levels of CREB, pri-miR-9-1 and mature miR-9 were detected in T98G and U251 cells infected with AD-shcreb or AD-shNC by quantitative RT-PCR (mean ± SD, n = 3). *, *P*<0.05, **, *P*<0.01, two-tailed unpaired Student's t test.

Because miR-9-1, miR-9-2 and miR-9-3 have all been predicted to be potential targets of the CREB transcription factor [26] and miR-9-2 was shown to be regulated by CREB during neuronal differentiation [25], we hypothesized that CREB could contribute to the up-regulation of miR-9 in glioma cells. Using bioinformatic prediction, we found two potential CREs in the 2-kb 5′ flanking sequence of miR-9-1 and one in the 5′ flanking sequence of miR-9-2 (Fig. 6A). ChIP-qPCR was utilized to test the binding capacity of CREB to these putative CREs in both T98G and U251 cells. The results showed that the CRE-a sequence in the 5′ flanking sequence of miR-9-1can be enriched by the CREB antibody (Fig. 6B), and knocking down CREB abolished this enrichment (Fig. 6C). Moreover, ectopic expression of CREB enhanced the transcriptional activity of the 5′ flanking sequence of miR-9-1, and mutation of CRE-a abolished the enhancive effect of CREB (Fig. 6D). For further confirmation of the regulation of miR-9-1 by CREB, we analyzed the effect of knocking down CREB on the expression levels of miR-9-1and mature miR-9 in T98G and U251 cells. Indeed, we found significant decreases in the levels of both pri-miR-9-1 and mature miR-9 (Fig. 6E), suggesting that CREB contributes to the high level of miR-9 by elevating the transcription of miR-9-1.

### Low glucose concentration induces the expression of miR-9 and a negative feedback minicircuitry coordinates the migration and proliferation of glioma cells

**Figure 7:**
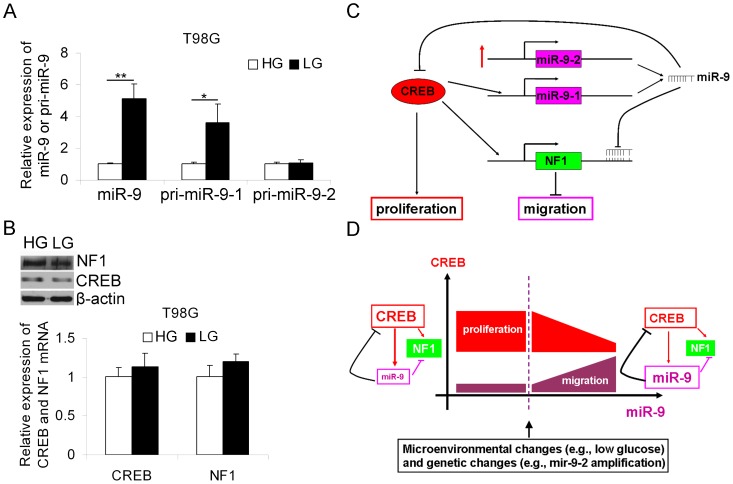
A low concentration of glucose induces the expression of miR-9. (A and B) T98G cells were maintained in DMEM/high glucose (4.5 g/L, HG) or DMEM/low glucose (1.0 g/L, LG) DMEM for 24 h. Cells were then harvested for RNA and protein extraction. The mRNA expression levels of miR-9, pri-miR-9-1, pri-miR-9-2, CREB and NF1 were determined by quantitative RT-PCR (mean ± SD, n = 3), and the protein levels of CREB and NF1 were detected by western blotting. (C) The negative feedback minicircuitry comprised of miR-9 and CREB. MiR-9 is highly expressed in glioma cells with amplification of the miR-9-2 gene copy number. The up arrow denotes genomic amplification. The transcription of miR-9-1 is regulated by CREB and miR-9 can directly target the 3′UTR of CREB, forming negative feedback minicircuitry. CREB inhibits the migration of glioma cells by elevating the expression level of NF1, whereas miR-9 promotes migration by directly targeting NF1 and CREB. In addition, miR-9 inhibits the proliferation of glioma cells by directly targeting the proliferation-promoting transcription factor, CREB. (D) The balance between miR-9 and CREB coordinates the migration and proliferation of glioma cells. The glioma cells with low levels of miR-9 and high CREB protein levels prefer to proliferate rather than migrate. As the glioma progresses, certain events, such as glucose reduction and mir-9-2 gene copy number amplification trigger the substantial increase of miR-9. By targeting migration-inhibitory CREB and NF1, miR-9 promotes the migration of glioma cells accompanied by proliferation repression.

One model of the “go or grow” phenomenon has reported that low glucose concentrations stimulate the migration of glioma cells [34]. Interestingly, we found that cells maintained in low glucose medium expressed significantly higher levels of mature miR-9 and pri-miR-9-1 but not pri-miR-9-2 (Fig. 7A), consistent with the migratory phenotype of glioma cells. Simultaneously, a low concentration of glucose led to a remarkable decrease in NF1 protein and a slight decrease in CREB protein level, while mRNA levels of NF1 and CREB were unaffected (Fig. 7B).

The results described above provide evidence of a regulatory network comprised of CREB, miR-9 and NF1 that modulates the migration and proliferation of glioma cells. As described in Fig. 7C, the pro-proliferative transcription factor CREB can directly regulate the expression of NF1 and miR-9-1. MiR-9, the microRNA product of miR-9-1 and miR-9-2, can directly target the 3′UTR of NF1 and CREB. These results suggest that the negative feedback minicircuitry consisting of CREB and miR-9 determines the glioma cells' phenotypes. As shown in Fig. 7D, the glioma cells with high expression of CREB and low- or moderate-level expression of miR-9 prefer to proliferate. In the progression of glioma, certain events, including miR-9-2 gene copy number amplification and microenvironmental changes, trigger the substantive expression of miR-9, and the balance between CREB and miR-9 might shift to pro-migratory and anti-proliferative. As a result, the glioma cells probably start to actively migrate, resulting in increased metastasis.

## Discussion

Because miR-9 is highly expressed in glioma cells, we deduced that it should contribute to the malignancy of glioma. In our study, we found a proliferation-inhibitory role for miR-9 by targeting CREB in glioma cells, which appears inconsistent with its high expression level. We also confirm a migration-enhancive role of miR-9, suggesting dual roles for miR-9 in modulating the proliferation and migration of glioma cells. Conversely, CREB, which has been previously identified as a proliferation enhancer of glioma cells [24], also functions as a migration inhibitor in glioma cells. CREB signaling was reported to positively regulate the migration of breast cancer and mesothelioma cells [23], and there have been no reports suggesting a similar migration-inhibitory function of CREB in human malignancies. We confirmed that CREB can positively regulate the expression of NF1 in glioma cells, which might explain the migration-inhibitory role of CREB. In addition to miR-9 and CREB, other genes have been shown to play dual roles in glioma cells. For example, under normal glucose conditions, elevated levels of miR-451 promote cell proliferation and decrease cell migration, while in a glucose-scarce environment, a decrease in miR-451 slows the proliferation and enhances the migration of glioma cells [32]. Additionally, carboxypeptidase E (CPE), a neuropeptide-processing enzyme, has a pro-proliferative and anti-migratory role in glioma cells [8]. This phenomenon of one gene playing opposite roles in proliferation and migration might be a common mechanism in glioma cells, although only a few examples can be adduced thus far.

In our study, we identified NF1, a well-known glioma suppressor, as a functional target of miR-9 in the regulation of glioma cell migration. Interestingly, the expression of NF1 is positively regulated by CREB. A similar manner of regulation of E2F1 has been discussed by O'Donnell et al., who found that MYC simultaneously activates E2F1 transcription and limits its translation through up-regulating E2F1-targeting miRNAs [33]. This type of two-tier regulation allows tight control of the expression levels of target genes and relevant cell signals. Because both CREB and miR-9 are highly expressed in glioma cells compared with normal glial cells, the NF1 protein level is determined by the balance between them. Moreover, in addition to directly targeting the NF1 3′UTR, miR-9 can indirectly repress the expression of NF1 by targeting its activator, CREB. In this study, our cell models are glioblastoma cell lines (U87MG, T98G and U251) with high expression levels of miR-9, partly due to copy number amplifications of the miR-9-2 gene. As a result, the balance shifts toward miR-9 and the expression of NF1 is restrained in these glioma cells.

Another interesting finding in our study is that miR-9 can target the 3′UTRs of CREB and NF1 that do not contain predicted miR-9 binding sites, as determined by the algorithms in common use. Inadvertently, we found that miR-9 can interact with the putative miR-182 binding sites on the 3′UTR of CREB and NF1, although miR-9 is not predicted to be a potential CREB/NF1-targeting miRNA by the three target prediction databases (TargetScan, PicTar and MiRDB). In a recent report, CREB was identified as a target of miR-182 in gastric cancer cells [27], which is also supported by our experimental data in glioma cells. In our study, we found that the miR-182 binding site is able to sufficiently interact with miR-9 to mediate a repressive effect. Mutation of this miR-182 binding site (TGCCAAA) abolishes the repressive activity of miR-9 on the CREB/NF1 3′UTR reporters. Utilizing bioinformatic algorithms to predict potential target genes that contain conserved 3′UTR sequences complementary to a seed region at the 5′ end of the miRNAs is the most common approach to identify miRNA target genes [34]. However, this bioinformatic approach is hampered by the fact that the existing algorithms have a high margin of error (some real target genes are not predicted) [35]. Lal et al. have shown that miR-24 can directly target E2F2, MYC and other cell-cycle genes via binding to “seedless” 3′UTR miRNA recognition elements [36]. Our results provide further evidence of the limitations of the conventional prediction algorithms. We hypothesize that the interaction of miR-9 with the putative miR-182 binding sites is a type of universal mechanism in glioma cells. In addition to CREB and NF1, previously established targets of miR-9 in glioma stem cells, such as CAMTA1 [18] and JAK1/2 [35], might contribute to the function of miR-9 in glioma cells, although further investigation will be required. Surprisingly, CAMTA1 was identified as a tumor suppressor in glioblastoma cancer stem cells [18], while JAK-STAT signaling was reported to promote gliomagenesis. Thus, the roles of miR-9 are complex.

In the theory of migration-proliferation dichotomy, migration and proliferation are mutually exclusive [6]. Under normal conditions, the cancer cells proliferate rapidly with very low migration rate. As the tumor size reaches a threshold, the exhaustion of diffusion-driven oxygen and local nutrition are insufficient to support further growth. The scarce environment and high mutation rates of tumor cells can then result in the emergence of highly motile tumor cells [37]. Interestingly, such “go or grow” phenomenon can be observed not only in cancer cells but also in macroscopical layers. For example, animals tend to increase their movement when they are near starvation. Under scarce environmental conditions, leaving for a new location with a plentiful food supply will benefit the survival of both the individual and species. It is believed that this “go or grow” strategy is evolutionarily ancient and phylogenetically widespread in nature [37].

The dual roles of miR-9 and CREB provide appropriate examples in support of the theory of migration-proliferation dichotomy. In our previous study, we found that the expression of CREB is not progressively increased, although it is still highly expressed in high-grade gliomas [24]. This highly expressed CREB supplies the tumor with an abundance of proliferation signals while inhibiting the spread of tumor cells. The inhibition of migration is actually propitious to cell proliferation. The increase in the migration enhancer miR-9, which is triggered by extracellular signaling or gene copy number amplifications, contributes to the motility of glioma cells. The inhibition of proliferation caused by highly expressed miR-9 also aids in the migration of glioma cells. Our study proposes a potential negative feedback minicircuitry comprising CREB and miR-9, where CREB contributes to the transcriptional activation of miR-9-1 and miR-9 represses the expression of CREB at the post-transcriptional level. The phenotypes of glioma cells (proliferative or migratory) are possibly determined by the balance of this feedback minicircuitry, ensuring that the glioma cells can properly react to environmental changes, such as glucose concentration, by coordinating proliferation and migration.

In summary, the present study proposes a novel feedback minicircuitry comprising CREB and miR-9 that coordinates the migration and proliferation of glioma cells. As summarized in Fig. 7C, we identified anti-proliferative and pro-migratory roles for miR-9, which can also target NF1, in glioma cells. In addition, the pro-proliferative transcription factor CREB was shown to regulate the expression of NF1 and inhibit the migration of glioma cells. Because the migration-proliferation dichotomy is a common mechanism during glioma progression, our findings provide evidence that one gene can play opposite roles in modulating glioma cell proliferation and migration. Importantly, our study suggests that the balance between CREB and miR-9 determines the “go or grow” status of glioma cells, furthering our understanding of the transition from proliferative to migratory phenotype during glioma progression.

## Materials and Methods

### Samples and cell lines

The four established human glioblastoma cell lines (U87MG, T98G, A172 and U251) were purchased from American Type Culture Collection (ATCC). The human cervical carcinoma cell line HeLa was obtained from the China Center for Type Culture Collection (Wuhan, China). The human normal glial cell line HEB was kindly provided by Dr. Guangmei Yan (Department of Pharmacology, Zhongshan School of Medicine, Sun Yat-Sen University, Guangzhou, China) [36]. HeLa, A172 and U251 cells were maintained in Dulbecco's Modified Eagle Medium supplemented with 10% fetal bovine serum (FBS), 5 mM L-glutamine, 100 U/ml penicillin and 100 mg/ml streptomycin. T98G and U87MG cells were maintained in Modified Eagle Medium supplemented with 1 mM sodium pyruvate and 1% NEAA, in addition to the usual FBS, L-glutamine and antibiotics. The HEB cell line was maintained in Dulbecco's Modified Eagle Medium with 1% NEAA and the normal FBS, L-glutamine and antibiotics.

### RNA extraction, cDNA synthesis and genomic DNA extraction

Total RNA was extracted from confluent plates of HeLa cells and normal human glial HEB cells using TRIzol (Invitrogen) according to manufacturer's protocol. cDNA was generated using the TranScript First-Strand cDNA Synthesis SuperMix (Transgen Biotech, China). To extract genomic DNA, cell pellets were lysed in cell lysis buffer (10 mM Tris-HCl (pH 8.0), 10 mM NaCl and 0.2% NP-40) for 10 min on ice. After centrifugation for 5 min at 2500×g (4°C), the pellets were resuspended in nuclear lysis buffer (50 mM Tris-HCl (pH 8.1), 10 mM EDTA and 1% SDS) for 10 min on ice. Genomic DNA was then purified from the nuclear lysate using the conventional phenol-chloroform extraction method.

### Cell transfection and infection

Plasmids, synthetic miRNA mimics or antagomirs and siRNAs were transfected into glioma cells using Lipofectamine 2000 according to the manufacturer's protocol. For infection, glioma cells were maintained in culture medium containing recombinant adenovirus (AD-shcreb or AD-shNC) at a final concentration of 1×10^7^ pfu/ml. After 12 h, the medium was replaced with complete medium, and the cells were maintained until the next experiments.

### ChIP-qPCR

For ChIP assay, approximately 1×10^7^ cells were harvested in medium and fixed with 1% formaldehyde. Glycine solution was added at a final concentration of 0.125 M to quench unreacted formaldehyde. Fixed cells were collected by spinning at 700×*g* for 5 min. ChIP experiments were performed using the EZ-ChIPTM Chromatin Immunoprecipitation Kit (Millipore, catalog #17-371) according to the manufacturer's protocol. The resulting ChIP products were used for quantitative real-time PCR.

### MTT and colony formation assays

Cell growth was assayed using the standard MTT method. Twenty-four hours after transfection, cells were maintained in serum-free medium, and the surviving cell number was determined using MTT. For colony formation assays, transfected or infected cells were plated in 12-well plates at 200 cells per well. After 10 to 14 days, cell colonies were stained and counted.

### Transwell migration and scratch wound healing assays

For transwell migration assays, transwell plates were purchased from Corning in a 24-well format with upper chamber inserts containing a membrane with an 8-µm pore size at the bottom (Corning 3422). Glioma cells were transfected with synthetic miRNA mimics, antagomirs or siRNAs or infected with AD-shcreb/AD-shNC. After 24 h, the transfected/infected glioma cells were maintained in serum-free medium for another 12 h and then resuspended at a final concentration of 5×10^5^ cells/ml in serum-free medium. One hundred microliters of the cell suspension was transferred to the upper chamber inserts. Another 600 µl of complete medium was loaded to the lower chambers. After 24 h, the remaining cells on the upper side of the membrane were removed and the membrane was stained with crystal violet, followed by microscopic observation. Staining was then removed with 33% (v/v) acetic acid solution and quantified by absorbance measurement (OD570). For scratch wound healing assays, when the transfected/infected glioma cells reached 80% confluence, a wound was created by scratching with a 200-µl pipette tip. After scratching, the detached cells were removed by washing twice, and the remaining cells were maintained in fresh medium. Photos were taken at 0, 24 or 48 h after scratching, and migration distance was calculated by measuring the width of the wound.

### Protein extraction and Western blotting

Total cellular protein was extracted using TNTE buffer (20 mM Tris-HCl (pH 7.5), 150 mM NaCl, 0.3% Triton X-100, and 5 mM EDTA) supplemented with protease inhibitors (2 µg/ml leupeptin, 2 µg/ml aprotinin, 2 µg/ml pepstatin and 2 µg/ml PMSF). Lysates were separated by 8% (or 10%) sodium dodecyl sulfate polyacrylamide gel electrophoresis and the gel was transferred onto a nitrocellulose membrane. The proteins were probed with rabbit anti-CREB (#9197, CST) or anti-NF1 (sc-67, SantaCruz) antibodies or a mouse anti-human β-actin antibody (A5441, Sigma).

### Quantitative real-time PCR

The expression levels of pri-miR-9-1, -2, and -3 as well as CREB and NF1 mRNAs were determined by real-time PCR using a SYBR-green-containing PCR kit (Takara) according to the manufacturer's instructions. Stem-loop RT-PCR for mature miR-9, miR-23a and miR-182 were performed as previously described [37]. To detect the gene copy numbers of the miR-9-1, miR-9-2 and miR-9-3 genes, genomic DNA was subjected to real-time PCR using primers specific for amplifying pre-miR-9-1, -2 or -3.

### miRNA ectopic expression and knockdown

Over-expression or knockdown of miRNAs was carried out by transfecting glioma cells with synthetic miRNA mimics or antagomirs (purchased from Invitrogen), respectively, at a final concentration of 50 nM. Over-expression and knockdown were assessed by quantitative RT-PCR.

### CREB and NF1 knockdown

CREB expression was silenced by adenovirus-mediated shRNA (target sequence: 5′-CAA TAC AGC TGG CTA ACA AT-3′). NF1 was knocked down by transfecting the glioma cells with specific siRNA against NF1 (target sequence: 5′- CTT CGG AAT TCT GCC TCT G -3′) at a final concentration of 50 nM. The efficiency of CREB and NF1 knockdown were evaluated by Western blotting and quantitative RT-PCR. The siRNA and adenovirus were purchased from GenePharma (Shanghai, China) and Genechem (Shanghai, China), respectively.

### Luciferase reporter assay

To test the interactions between the 3′UTR of CREB/NF1 and miR-9 in T98G, 100 ng of each of the 3′UTR-LUC reporter was cotransfected with 50 ng phRL-TK (Renilla Luciferase) for normalization and 50 nM synthetic miRNA mimics (miR-9, 23a and 182)/miRNA antagomirs (anti-9, 23a, 182) or control miRNA mimics (miR-NC)/control antagomirs (anti-NC). After 48 h, lysates of 293ET or T98G cells from all treatment groups were collected using Passive Lysis Buffer (Promega). Firefly luciferase activity was analyzed relative to Renilla luciferase activity in the same sample using a dual luciferase reporter assay system (Promega). Luminescence was measured using the GloMax multi-detection system (Promega). Three independent experiments were performed and assayed in quadruplicate per group.

### Computational prediction

Three target prediction databases (TargetScan, PicTar and MiRDB) were used to analyze the interactions between miR-9 and CREB/NF1 3′UTRs. Putative CRE elements within the 2 kb 5′ flanking sequence of miR-9-1 and miR-9-2 were predicted with TFSEARCH (http://www.cbrc.jp/research/db/TFSEARCH.html). The alignment of the mammalian CREB and NF1 3′UTRs and the conservation track of the NF1 promoter region was obtained from the UCSC Genome browser (http://genome.ucsc.edu/).

## Supporting Information

Figure S1 MiR-9 knockdown slows the wound healing of glioma cells. Glioma cells (U87MG, T98G and U251) were transfected with miR-9 antagomirs (anti-9) or control antagomirs (anti-NC). Cells were subjected to scratch wound healing assays upon reaching 80% confluence. Representative photographs are shown.
(DOC)

Figure S2 MiR-9 targets CREB in a miR-182-independent manner. (A) The expression levels of miR-182 in HeLa, HEB and four glioma cell lines were determined by quantitative real-time PCR (mean ± SD, n = 3). (B) T98G cells were transfected with miRNA antagomirs (anti-9, anti-23a or anti-182) or control (anti-NC). After 48 h, the expression levels of the miRNAs were determined by quantitative RT-PCR (mean ± SD, n = 3).
(DOC)

Figure S3 NF1 expression in glioma cell lines and the role of NF1 in glioma cell proliferation. (A and B) The mRNA and protein levels of NF1 in HeLa, HEB and glioma cell lines (U87MG, T98G, A172 and U251) were determined by quantitative RT-PCR and Western blotting, respectively. (C) The MTT method was employed to determine the effects of NF1 siRNA-mediated knockdown on T98G and U251 cell growth and survival, as determined by absorbance measurement (570 nm) (mean ± SD, n = 4). (D) T98G and U251 cells transfected with siRNA against NF1 were subjected to colony formation assays. Cell colonies were counted and plotted (mean ± SD, n = 4).
(DOC)

Figure S4 Over-expressing miR-9 and knocking down NF1 promote the migration of U251 cells. (A) U251 cells were transfected with synthetic miR-9 mimics (or miR-NC) and siRNA targeting NF1 (or control siRNA, siNC). After 48 h, total cellular protein was extracted and Western blotting was performed to detect the protein level of NF1. (B) The transfected U251 cells were also subjected to transwell migration assays. Representative photographs are shown. **, P<0.01, two-tailed unpaired Student's t test.
(DOC)

Figure S5 Knocking down CREB stimulates the migration of glioma cells. The glioma cells (U87MG, T98G and U251) were infected with AD-shcreb or AD-shNC. After reaching confluence, the cells were subjected to scratch wound healing assays. Representative photographs are shown at the top. The migration distances of the glioma cells were determined by measuring the wound widths at 0 and 24 h (mean ± SD, n = 5). *, P<0.05; ***, P<0.001, two-tailed unpaired Student's t test.
(DOC)

Figure S6 CREB directly regulates NF1. (A) The mammalian alignment showing a conserved CRE in the proximal promoter of the NF1 gene. The conservation track was obtained from the UCSC genome browser. (B) The promoter region containing a wild type (TGACGTCA) or mutated CRE (TGCGTACA) was inserted upstream of the luciferase cassette. (C) The luciferase reporter constructs were co-transfected with the CREB expression plasmid or control vector, and the normalized luciferase activity was determined 48 h later (mean ± SD, n = 4). *, P<0.05, two-tailed unpaired Student's t test.
(DOC)
