# Supplementary figures and images for: The CREB-miR-9 Negative Feedback Minicircuitry Coordinates the Migration and Proliferation of Glioma Cells
Source: PLoS One. 2012 Nov 20;7(11):e49570. doi: 10.1371/journal.pone.0049570 (PMC3502497; doi:10.1371/journal.pone.0049570)

**Figure S1**


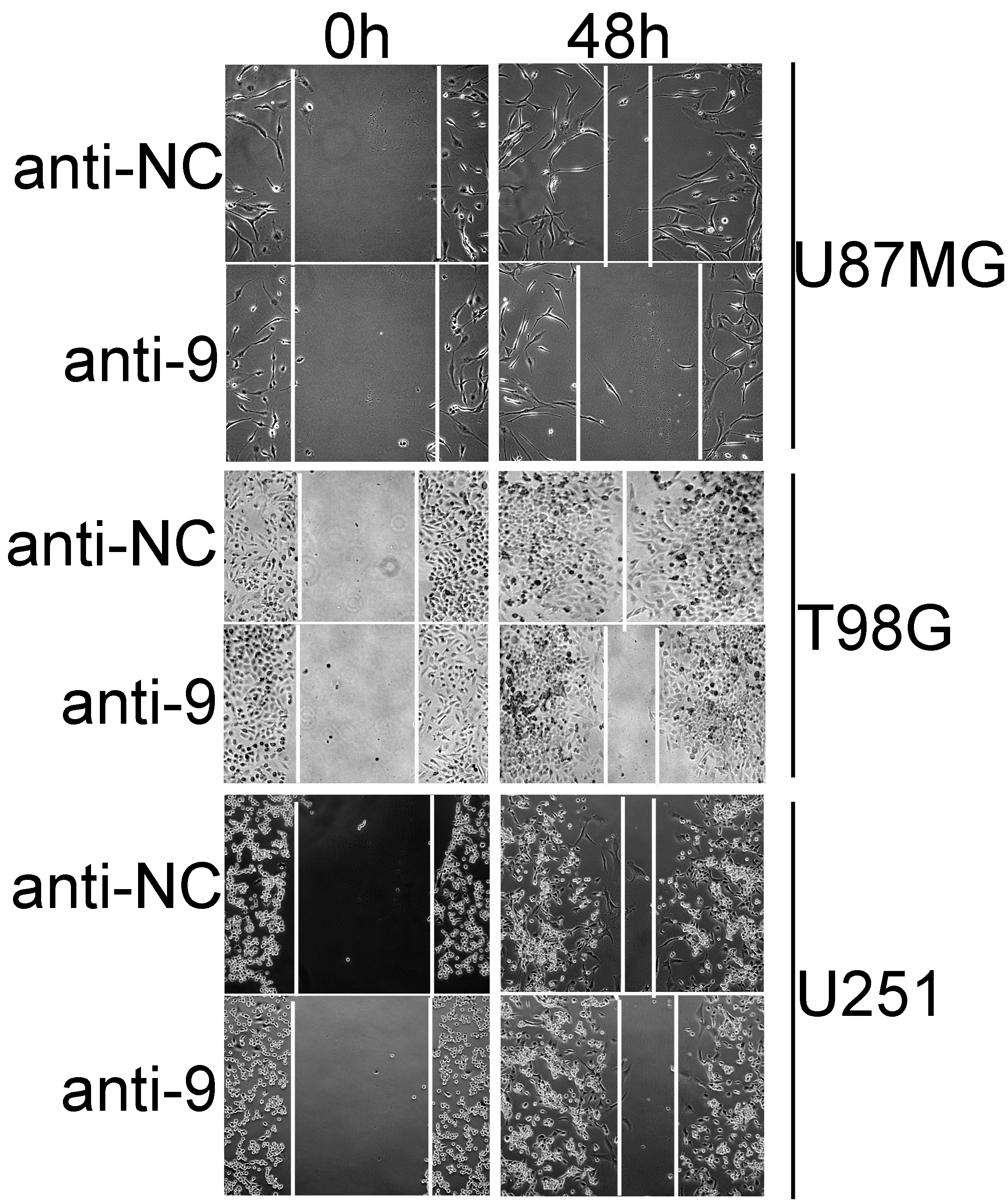

Supplement: Figure S1 — MiR-9 knockdown slows the wound healing of glioma cells. Glioma cells (U87MG, T98G and U251) were transfected with miR-9 antagomirs (anti-9) or control antagomirs (anti-NC). Cells were subjected to scratch wound healing assays upon reaching 80% confluence. Representative photographs are shown. (DOC) [file pone.0049570.s001.doc]

**Figure S2**

**A B**


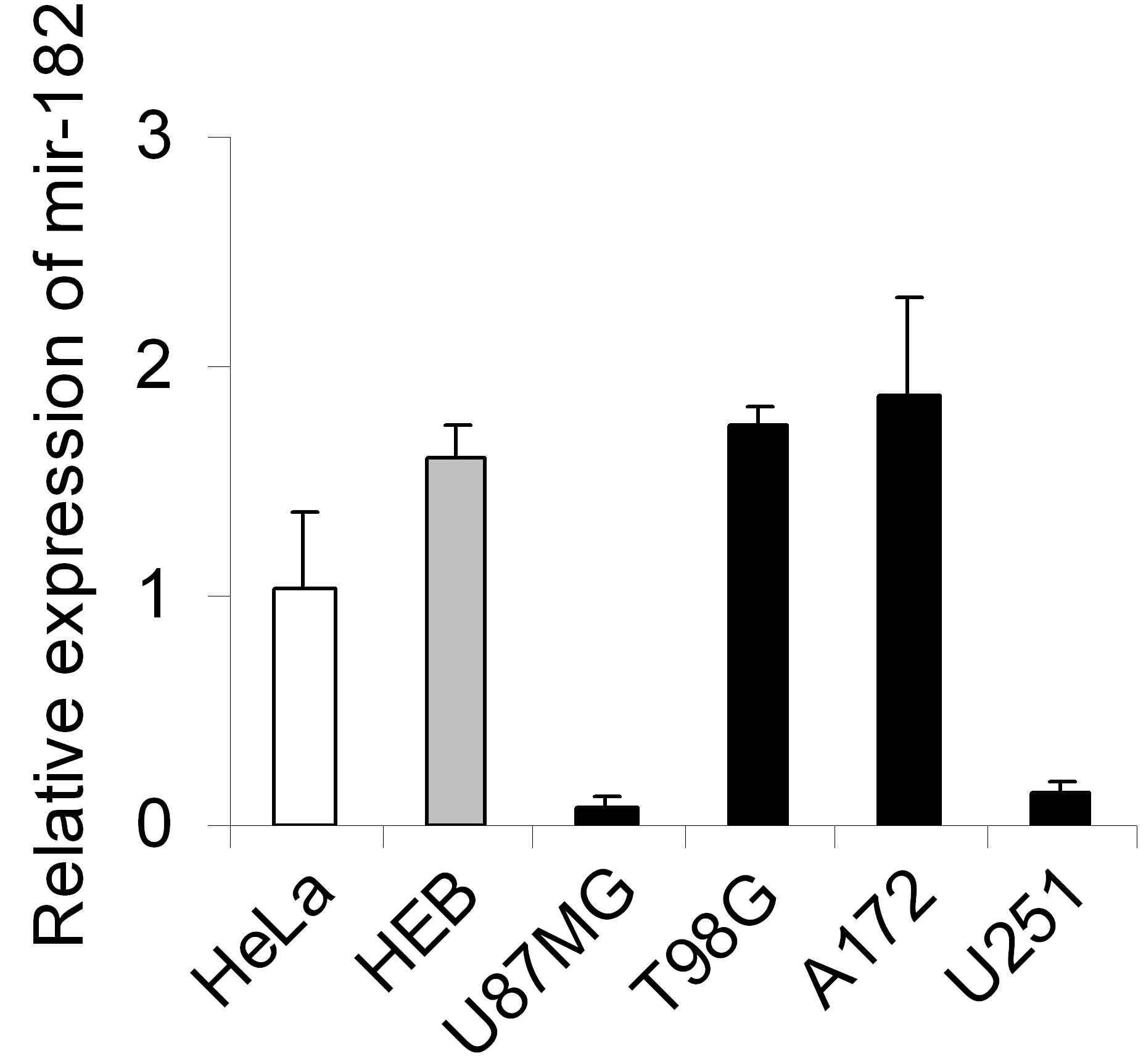

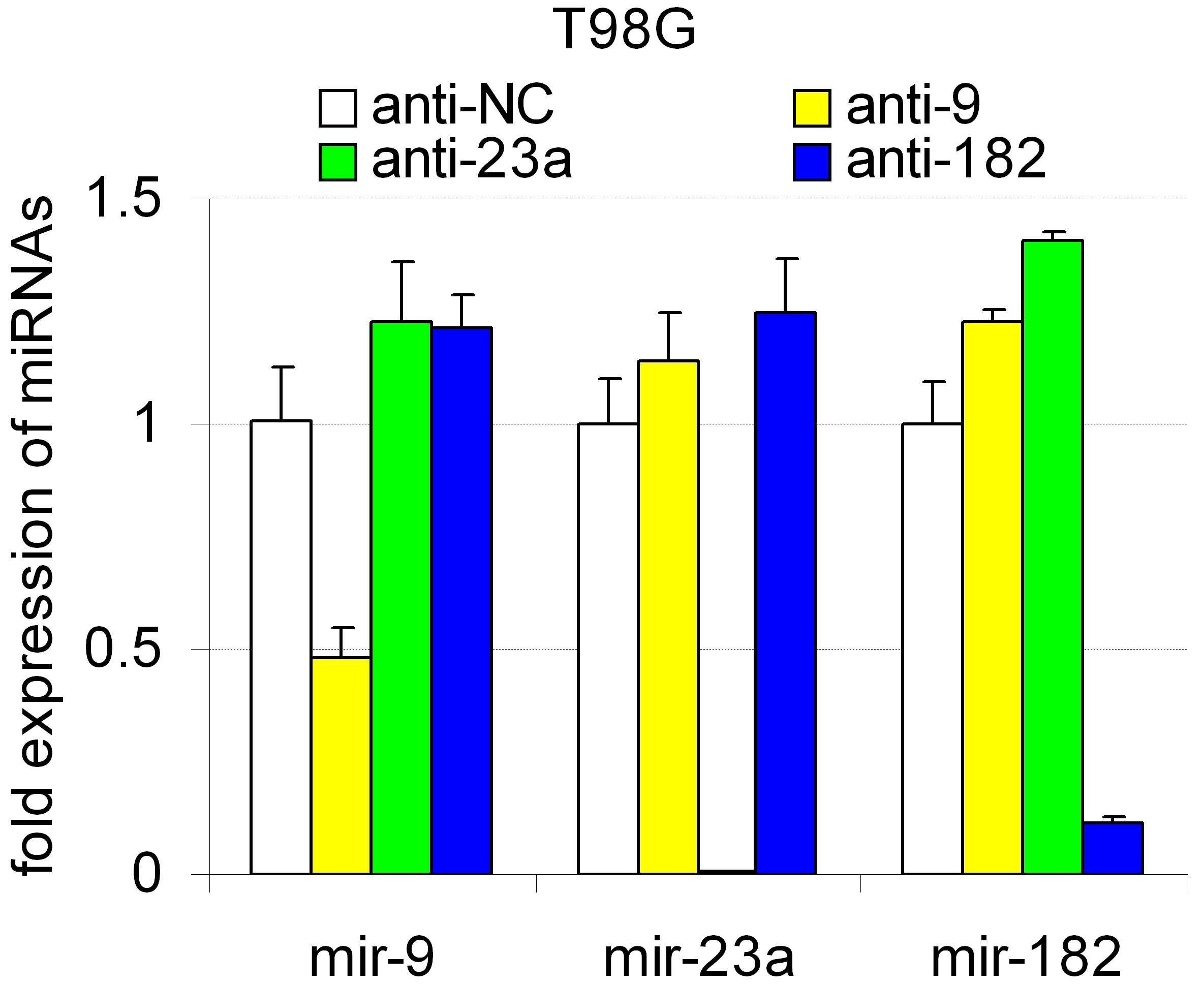

Supplement: Figure S2 — MiR-9 targets CREB in a miR-182-independent manner. (A) The expression levels of miR-182 in HeLa, HEB and four glioma cell lines were determined by quantitative real-time PCR (mean ± SD, n = 3). (B) T98G cells were transfected with miRNA antagomirs (anti-9, anti-23a or anti-182) or control (anti-NC). After 48 h, the expression levels of the miRNAs were determined by quantitative RT-PCR (mean ± SD, n = 3). (DOC) [file pone.0049570.s002.doc]

**Figure S3**


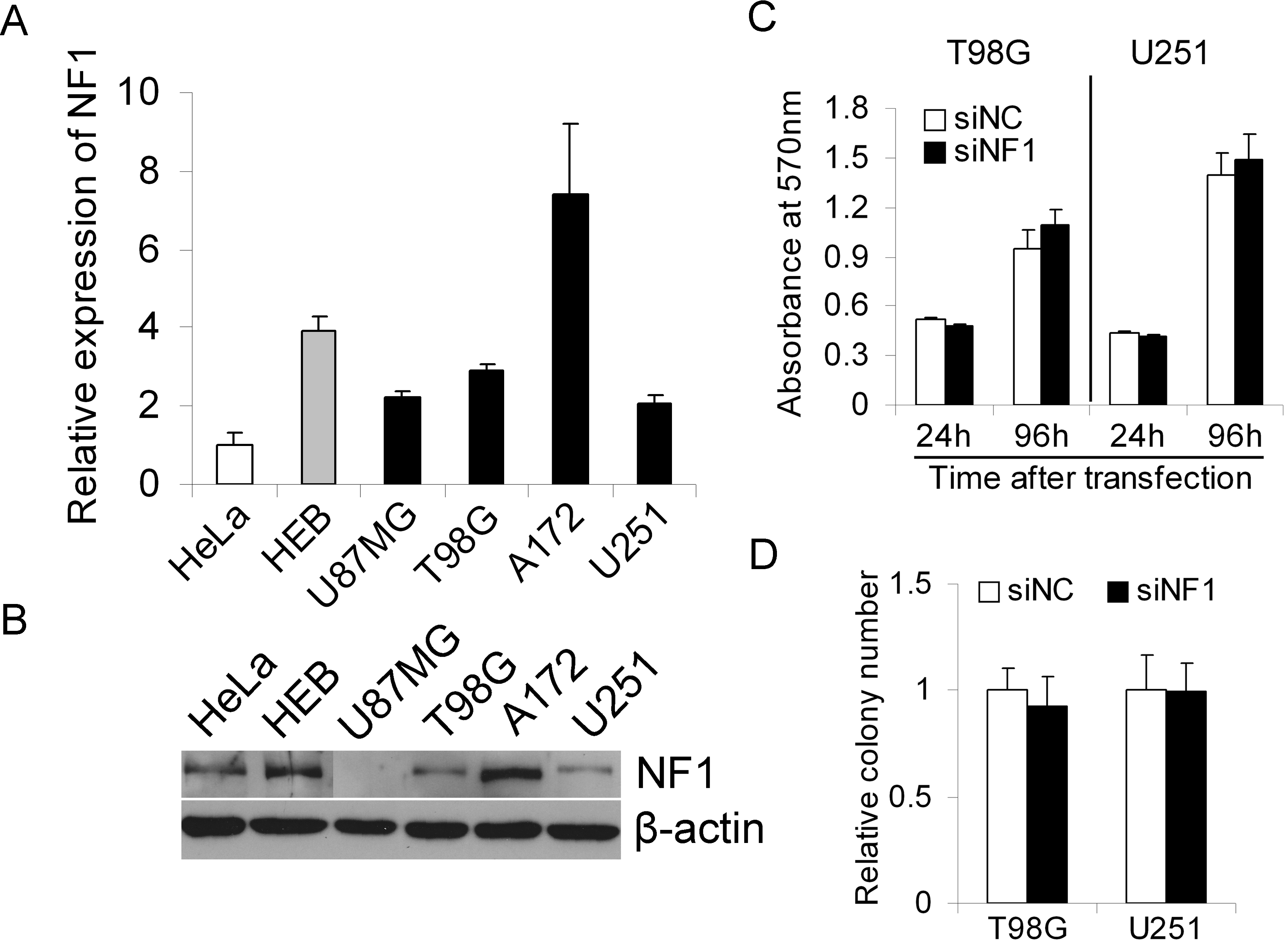

Supplement: Figure S3 — NF1 expression in glioma cell lines and the role of NF1 in glioma cell proliferation. (A and B) The mRNA and protein levels of NF1 in HeLa, HEB and glioma cell lines (U87MG, T98G, A172 and U251) were determined by quantitative RT-PCR and Western blotting, respectively. (C) The MTT method was employed to determine the effects of NF1 siRNA-mediated knockdown on T98G and U251 cell growth and survival, as determined by absorbance measurement (570 nm) (mean ± SD, n = 4). (D) T98G and U251 cells transfected with siRNA against NF1 were subjected to colony formation assays. Cell colonies were counted and plotted (mean ± SD, n = 4). (DOC) [file pone.0049570.s003.doc]

**Figure S4**


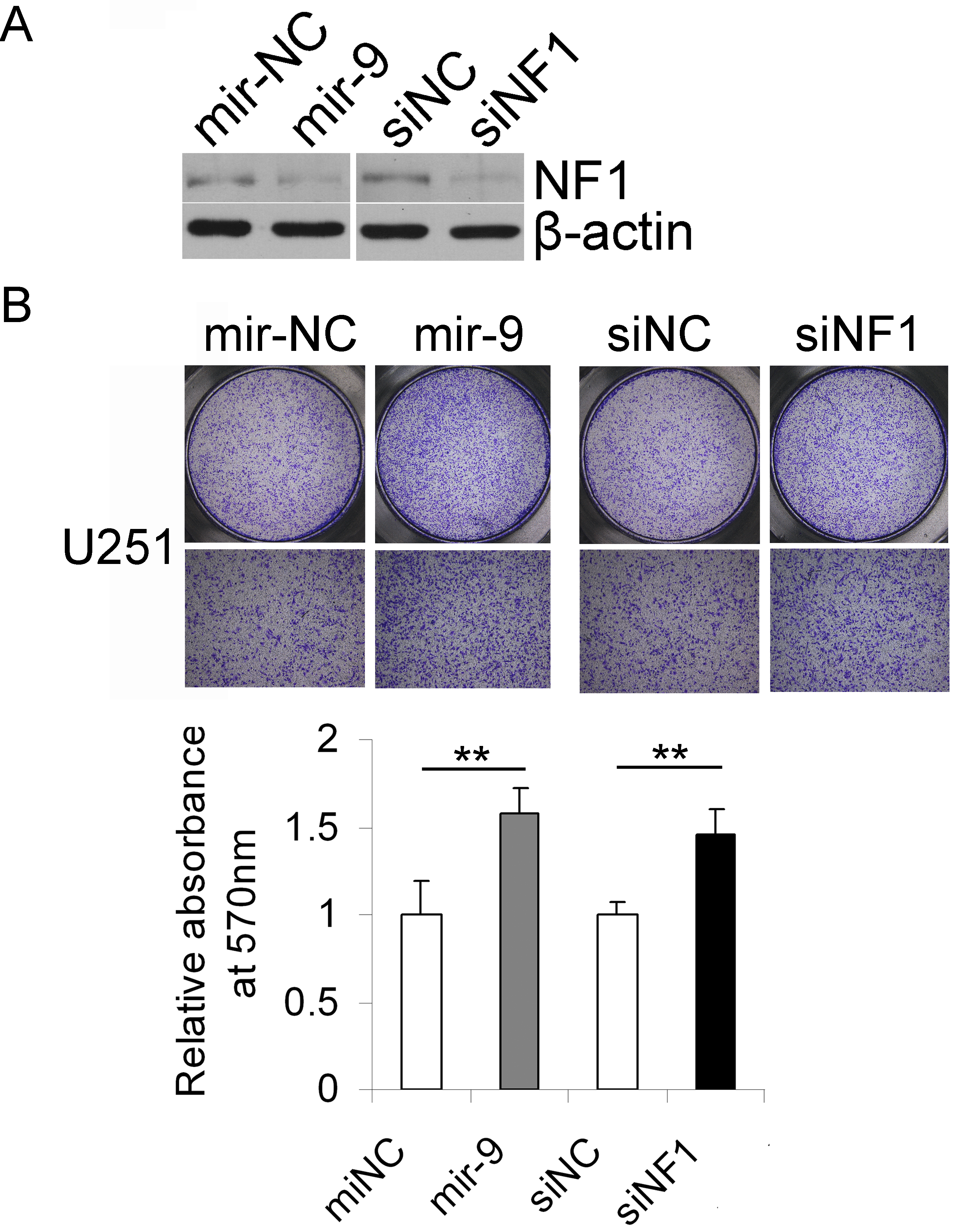

Supplement: Figure S4 — Over-expressing miR-9 and knocking down NF1 promote the migration of U251 cells. (A) U251 cells were transfected with synthetic miR-9 mimics (or miR-NC) and siRNA targeting NF1 (or control siRNA, siNC). After 48 h, total cellular protein was extracted and Western blotting was performed to detect the protein level of NF1. (B) The transfected U251 cells were also subjected to transwell migration assays. Representative photographs are shown. **, P<0.01, two-tailed unpaired Student's t test. (DOC) [file pone.0049570.s004.doc]

**Figure S5**


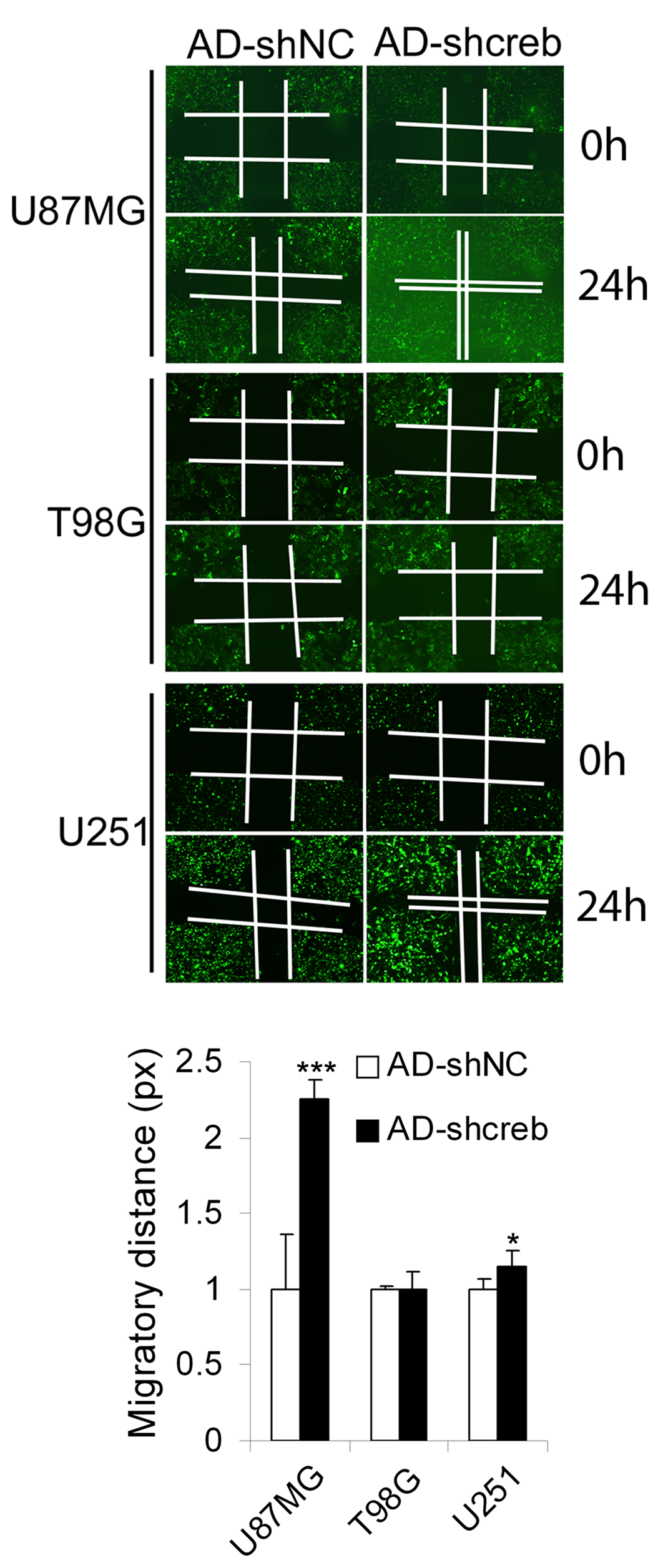

Supplement: Figure S5 — Knocking down CREB stimulates the migration of glioma cells. The glioma cells (U87MG, T98G and U251) were infected with AD-shcreb or AD-shNC. After reaching confluence, the cells were subjected to scratch wound healing assays. Representative photographs are shown at the top. The migration distances of the glioma cells were determined by measuring the wound widths at 0 and 24 h (mean ± SD, n = 5). *, P<0.05; ***, P<0.001, two-tailed unpaired Student's t test. (DOC) [file pone.0049570.s005.doc]

**Figure S6**


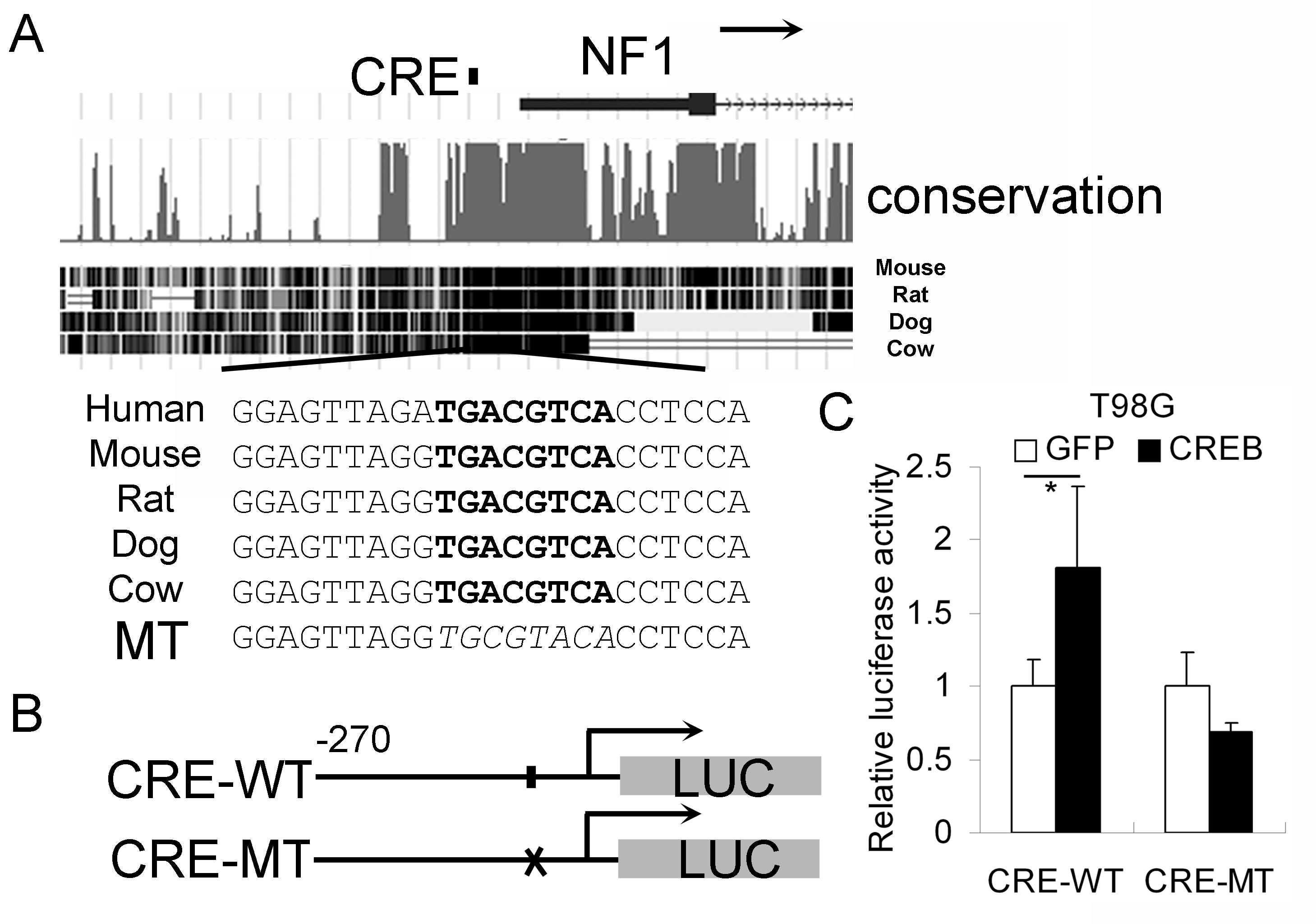

Supplement: Figure S6 — CREB directly regulates NF1. (A) The mammalian alignment showing a conserved CRE in the proximal promoter of the NF1 gene. The conservation track was obtained from the UCSC genome browser. (B) The promoter region containing a wild type (TGACGTCA) or mutated CRE (TGCGTACA) was inserted upstream of the luciferase cassette. (C) The luciferase reporter constructs were co-transfected with the CREB expression plasmid or control vector, and the normalized luciferase activity was determined 48 h later (mean ± SD, n = 4). *, P<0.05, two-tailed unpaired Student's t test. (DOC) [file pone.0049570.s006.doc]
